# Relationship between postural changes in pretracheal tissue depth and body mass index

**DOI:** 10.1097/MD.0000000000042110

**Published:** 2025-05-23

**Authors:** Seong Wook Park, Seok Ran Yeom, Wook Tae Yang, Won Woong Tae, Ji Ho Ryu, Mose Chun

**Affiliations:** a Department of Emergency Medicine, Pusan National University Hospital, Busan, Korea; b Department of Emergency Medicine, School of Medicine, Pusan National University, Busan, Korea; c Department of Emergency Medicine, Pusan National University Yangsan Hospital, Busan, Korea.

**Keywords:** body mass index, trachea, tracheostomy, ultrasound

## Abstract

**Background:**

The most common serious complication associated with tracheostomy is unintentional displacement of the tracheostomy tube. Therefore, it is important to predict the probability of the tube displacement. Obese pretracheal soft tissue thickness related to obesity is the most likely cause of tracheal tube displacement. An increase in pretracheal tissue depth resulting from postural changes may reduce the intratracheal length of the tube and increase the risk of tube displacement. This study analyzed the relationship between postural differences in the pretracheal tissue depth and body mass index (BMI).

**Methods:**

This prospective clinical trial included 100 patients who were enrolled between June 2019 and November 2020. An ultrasound probe was used to measure the pretracheal tissue depth in neutral and extended neck positions. Neck extension was achieved by placing a pillow on the patient’s back. Differences in the pretracheal tissue depth were calculated by subtracting the extended neck depth from the neutral neck depth. The patients’ BMI were calculated using weight and height data.

**Results:**

Differences in pretracheal tissue depth were correlated with neutral depth (*r* = 0.721) and BMI (*r* = 0.436). The BMI and pretracheal tissue depth were moderately, positively, and linearly correlated (neutral position: *r* = 0.574, extended position: *r* = 0.486).

**Conclusions:**

Postural differences in the pretracheal tissue depth measured using ultrasonography are correlated with BMI and may be used to predict the risk of tracheostomy tube displacement.

## 1. Introduction

Obesity, defined as a body mass index (BMI) >30 kg/m^2^, is increasing, with a prevalence of 42% among adults in the United States.^[1]^ Obesity is associated with complications of tracheostomy.^[2–5]^ Serious perioperative complications such as airway loss and tracheoesophageal injury are more common in obese patients.^[4]^

Tracheostomy is one of the most common surgical procedures performed in patients admitted to the intensive care unit. The indications for tracheostomy include upper airway obstruction, head or neck trauma, and persistent respiratory failure. Several tracheostomy-related complications have been reported, including unintentional tube displacement, which is a serious complication that occurs in approximately 1.5% of tracheostomy procedures.^[6]^ When the tube is displaced, the resulting loss of airway patency leads to hypoxia. Additionally, subcutaneous emphysema and pneumothorax are induced when positive-pressure ventilation is continued after tube displacement,^[7]^ which may lead to hypoxic brain injury and cardiac arrest without immediate intervention. Factors associated with tube displacement include tube length, neck thickness, postoperative swelling, and poorly secured tubes.^[6]^ Increased pretracheal soft tissue thickness, which decreases the intratracheal length of the tracheal tube, is the most likely cause of tube displacement.^[4,5,8–10]^ Postural changes that increase the pretracheal tissue depth may decrease the intratracheal length of the tube, leading to tube displacement. This study explored the relationship between the pretracheal tissue depth at different neck positions and BMI.

## 2. Materials and methods

This prospective clinical trial was conducted between June 2019 and November 2020. This study was approved by the Pusan National University Hospital Institutional Review Board (approval number 1608-007-044). Informed consent was obtained from the patient for the publication of this case report. Written informed consent was obtained for publication of individual data (sex, age, body weight, and height). All the patients included in this study were admitted to the emergency department of our hospital. Patients <18 years of age and those with prior neck surgery or trauma, cervical spine disease, or inability to withstand neck extension were excluded from the study.

**Figure 1.**
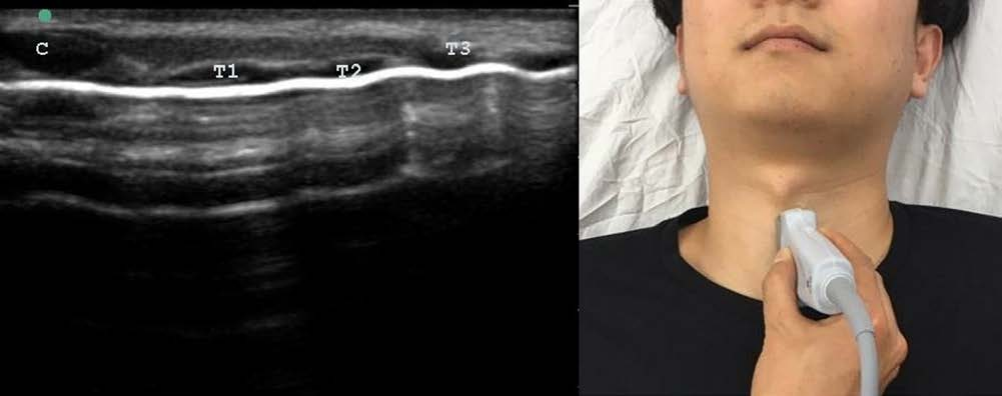
Parasagittal view at the trachea. C = cricoid cartilage, T1-T3 = first-third tracheal cartilage.

**Figure 2.**
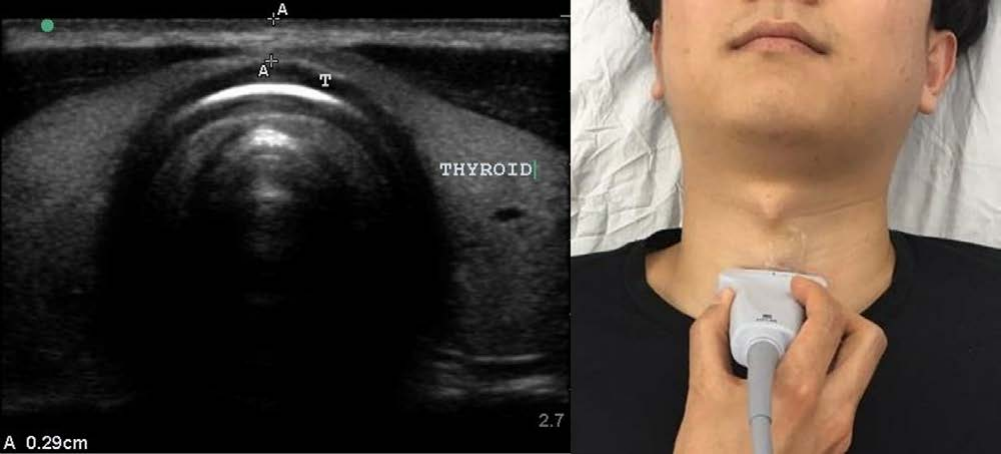
Transverse view at the trachea. The distance between A and A represents the pretracheal tissue depth. In this image, A–A was 0.29 cm. T = tracheal cartilage.

A linear ultrasonography probe (M-Turbo, 6–13 Hz; FUJIFILM Sonosite) was used to measure the pretracheal tissue depth at the second and third tracheal cartilages in neutral and extended neck positions in the parasagittal and transverse planes (Figs. 1 and 2). Neck extension was achieved by placing a pillow on the patient’s back. Differences in the pretracheal tissue depth were calculated by subtracting the pretracheal tissue depth during neck extension from that when the neck was in a neutral position. Weight and height data were used to calculate BMI.

All analyses were performed using SPSS (version 14.0; SPSS Inc., Chicago, IL). Categorical variables are presented as frequencies and percentages, and continuous variables are presented as means and standard deviations or medians and interquartile ranges, as appropriate according to the normality of the distribution. Pearson’s correlation analysis was used to determine correlations between the variables. Statistical significance was set at *P* < .05.

## 3. Results

A total of 100 patients (66 men and 34 women) were enrolled in this study. The mean patient age was 53.64 ± 17.69 years, the mean height was 166.12 ± 7.61 cm, the mean weight was 64.16 ± 10.83 kg, and the mean BMI was 23.20 ± 3.38 kg/m^2^ (Table 1). Postural differences in pretracheal tissue depth correlated with neutral depth (*r* = 0.721) and BMI (*r* = 0.436; Table 2). BMI and pretracheal tissue depth were moderately, positively, and linearly correlated (neutral pretracheal tissue depth, *r* = 0.574; extended pretracheal tissue depth, *r* = 0.486; Table 3).

**Table 1:** Patient characteristics.

Ultrasound measure (mm)	
PTDneu	8.29 ± 2.97
PTDext	6.80 ± 2.16
PTDdf (PTDneu-PTDext)	0.95(0.5–2.08)
PTDdf ratio (PTDneu-PTDext/PTDneu, %)	16.07 ± 11.97
Age (yr)	53.64 ± 17.69
Weight (kg)	64.16 ± 10.83
Height (cm)	166.12 ± 7.61
BMI (kg/m^2^)	23.20 ± 3.38

Values are presented as mean ± standard deviation or median (interquartile range).

BMI = body mass index, PTDdf ratio = pretracheal tissue depth difference ratio, PTDext = pretracheal tissue depth in the neck extension posture, PTDneu = pretracheal tissue depth in the neutral neck posture.

**Table 2:** Pearson’s correlation analysis between pretracheal tissue depth difference and other variables.

Variables	Correlation coefficient (*r*)	*P* value
Age (yr)	0.131	.193
BMI (kg/m^2^)	0.436	<.001
Weight (kg)	0.193	.055
Height (cm)	−0.32	.001
PTDneu (mm)	0.721	<.001
PTDext (mm)	0.294	.003
PTDdf ratio (%)	0.907	<.001

BMI = body mass index, PTDdf = pretracheal tissue depth difference, PTDext = pretracheal tissue depth in neck extension posture, PTDneu = pretracheal tissue depth in neutral neck posture.

**Table 3:** Pearson’s correlation analysis between body mass index and other variables.

Variables	Correlation coefficient (*r*)	*P* value
Age (yr)	0.084	.409
Weight (kg)	0.842	<.001
Height (cm)	−0.33	.748
PTDdf (mm)	0.436	<.001
PTDneu (mm)	0.574	<.001
PTDext (mm)	0.486	<.001
PTDdf ratio (%)	0.273	.006

PTDdf ratio = pretracheal tissue depth difference ratio, PTDext = pretracheal tissue depth in extended neck posture, PTDneu = pretracheal tissue depth in neutral neck posture.

## 4. Discussion

Tracheostomy is typically performed on a patient’s neck in extension. However, patients who have undergone tracheostomy routinely assume different neck postures, including neutrality and rotation, after the procedure. Therefore, pretracheal tissue thickness was measured in the neutral and extended positions in this study. It was hypothesized that postural changes increase pretracheal tissue depth, decrease intratracheal tube length, and induce tube displacement.

Clinically, it is important to predict the risk of tube displacement and to select an appropriate tube length. However, tube selection is often guided by the surgeon’s experience, qualitative evaluation of the patient’s body habitus, and tube fit during the procedure. Risk factors for tube displacement include tube length, neck thickness, postoperative swelling, and poorly secured tubes.^[6]^ With the exception of how secured the tube is, these risk factors are associated with a short tube length and thick pretracheal tissue depth. Neck thickness is often associated with obesity, and the complication rates of tracheostomy are higher in patients with obesity.^[4,5]^ The risk of serious complications of percutaneous tracheostomy was reported to be 5 times higher in patients with obesity in a previous study.^[4]^ Another study reported that the skin-to-trachea distance was a better predictor than BMI for tracheostomy dislodgement.^[11]^ A significant correlation between pretracheal tissue depth and the need for an extended-length tracheostomy tube was reported in a previous retrospective study of computed tomography images of patients who underwent tracheostomy.^[12]^ However, computed tomography is not a practical method to measure the pretracheal thickness.

Ultrasound has been used to examine the anatomy of the neck to guide invasive procedures, such as cricothyroid membrane cannulation and percutaneous tracheostomy, and to confirm endotracheal tube position.^[13–16]^ Ultrasound is commonly used in the intensive care unit because of its portability, cost-effectiveness, and lack of radiation. Szeto et al first reported the use of ultrasonography to measure the pretracheal tissue depth.^[10]^ The previous group determined if the anthropometric measurements correlated with the ultrasonographic measurements of the neck in the supine position. They also reported that waist circumference in the standing position and arm/neck circumference in the supine position correlated with pretracheal tissue depth measured via ultrasound. Another previous study suggested that ultrasound-guided measurements of anterior neck soft tissue thickness, BMI, and neck circumference could predict the difficulty of intubation in patients with obesity.^[17]^

This is the first study to analyze postural differences in the pretracheal tissue depth. These differences are correlated with pretracheal tissue depth in the neutral position and BMI, which are known risk factors for tube displacement. Therefore, postural differences in the pretracheal tissue depth may be used to predict the risk of tracheostomy tube displacement.

This study had several limitations. First, the small sample size of 100 patients may have limited the generalizability of the findings. Larger sample sizes are generally required to improve the statistical power and reliability. Second, the exclusion criteria, omitting patients aged <18 years and those with certain medical conditions, may limit the applicability of the findings to a broader population, especially because tracheostomies are performed across diverse patient groups. Third, the study did not assess patient outcomes such as prognosis and complications related to tracheostomy. Without data on actual tube displacements or associated complications, it was difficult to determine the clinical significance of the observed correlations. Lastly, the study lacked a clearly defined cutoff value for pretracheal tissue depth, leaving the clinical relevance of these measurements unclear. This uncertainty may affect the practical application of the findings in clinical decision-making.

## 5. Conclusion

Postural changes in pretracheal tissue depth are correlated with pretracheal tissue depth in the neutral position and BMI, which are known risk factors for tube displacement. Therefore, postural differences in the pretracheal tissue depth may be used as a predictor of the risk of tracheostomy tube displacement.

## Author contributions

**Conceptualization:** Ji Ho Ryu.

**Data curation:** Won Woong Tae, Mose Chun.

**Formal analysis:** Ji Ho Ryu.

**Investigation:** Wook Tae Yang.

**Methodology:** Won Woong Tae, Mose Chun.

**Supervision:** Seok Ran Yeom.

**Validation:** Wook Tae Yang.

**Writing – original draft:** Seong Wook Park.

**Writing – review & editing:** Seok Ran Yeom.
